# Activity system, schizotypal personality, and mentalization: A study between halted activity and COVID-19 conducted in Henan, China

**DOI:** 10.3389/fpubh.2022.930842

**Published:** 2022-08-09

**Authors:** Mohamad El Maouch, Yile Wang, Zheng Jin, Timothy Tamunang Tamutana, Kaibin Zhao, Yu Liu

**Affiliations:** ^1^Henan International Joint Laboratory of Psychological Data Science, Zhengzhou Normal University, Zhengzhou, China; ^2^Department of Journalism Studies, Faculty of Social Sciences, University of Sheffield, Sheffield, United Kingdom; ^3^Department of Psychology, University of California, Davis, Davis, CA, United States

**Keywords:** COVID-19, pandemic lifestyle, schizotypy, mentalization, stress, activity system, life narrative disturbance

## Abstract

The pandemic-related lifestyle has potentially imposed crucial disturbances on daily and long-term activities, which, in turn, were associated with thought disturbance. This study investigates how the characteristics of the activity system during pandemic-related restrictions are associated with other psychomental aspects. By focusing on PTSD, mentalization, and schizotypal personality, and by inquiring about the main components of the activity system of 852 college students (Zhengzhou, Henan, China)- including the goals orienting their activity, goals' terms and types, the motivation levels and sources, the activity type and engagement time, the flow of the activity, and how due to pandemic lifestyle-results revealed that the activity system's components have significant associations with PTSD, reflective function, and schizotypal traits. Additionally, some of the activity system's elements have a significant predictive role regarding schizotypal traits. The study considered that the life narrative during the pandemic has been disturbed; hence, this may have a crucial effect on mind coherence. Additionally, the outcomes from the pandemic context will support mental health interventions in other similar contexts where the life narrative is severely affected.

## Introduction

Although statistics are not accurate due to low social engagement in surveys, reportedly only 40% of the member states in the World Health Organization (WHO) have compiled mental health data in their general health statistics. Of this percentage, approximately 800 million people are living with mental health disorders, which showed an increase of up to 250% between 1990 and 2017. Schizophrenia affects approximately 20 million people worldwide, with an increase of 166% from 12 million in 1990 (1–6). Furthermore, as in any emergency and other infectious diseases outbreaks, COVID-19 as a global pandemic imposed crucial changes on our lifestyle, including fears, worry, experiencing loss of own and other lives, separation from beloved relatives, being isolated and quarantined, loss of employment and income, haltering daily activity, social stigma, discrimination of infected individuals, and uncertainty about the future; hence, reducing mental health stability, and leading to stress, depression, anxiety, distress, coping strategies, and insomnia (7–11).

As in previous outbreaks (e.g., 2003-SARS), and along with the economic crisis outcomes since 2008 (e.g., unemployment, social, and political disturbances), several studies have investigated influencing factors such as demographic information, living area, having relatives or acquaintances diagnosed with COVID-19, the amount of exposure and the access to accurate COVID-19 information from media and other resources, social support, prior mental health problem, educational status. It is found that people in quarantine might face anger, loneliness, boredom, and various psychiatric morbidities, including psychomotor excitement, panic attacks, delirium, psychotic symptoms, and suicidality [see, e.g., (7, 10, 12–17)].

Furthermore, investigating the severe forms of the pandemic's outcomes is crucial for developing suitable intervention tools, by promoting and implementing mental health and psychological assessment, counseling, support, and treatment through multidisciplinary mental health policies, e.g., psychiatric teams, clinical psychologists, hotlines, and online platforms, especially that till now, countries like China are still experiencing COVID-19-related lifestyle, including restrictions, social distancing, testing, and reporting to designated authorities, is ongoing for around 3 years (7, 10).

The causal origins of schizophrenia as a severe outcome are still under ongoing debate. For mainstream currents, it is the interaction among genetics-environmental factors (5). Also, the different explanations of schizophrenia do not reflect a continuum in the research context, and parallelism is noticed (12). Some focus on biochemical factors such as the genetic level and the disturbance of the brain's chemistry and liaison, and support pharmacology and antipsychotics (18, 19), despite “less definite” genetic components proved (20), and the indefinite or negative effect of antipsychotics on the quality of life (21–23). Although they do not have consistent effects on overall social functioning and pervasive positive and negative symptoms, others focus on familial intervention, psychoanalysis, cognitive remediation, and cognitive-behavioral therapy (22, 24). However, social intervention through social integration and employment, and reducing discrimination and stigma, showed significant results (24–26). Overall, addressing the causal explanation of psychosis and schizophrenia is still problematic, related more to the after-diagnosis, and is only partly dedicated to preventive purposes.

Therefore, early detection is required. Developing schizophrenia is considered to be harbored in a latent personality organization conceptualized through the term “Schizotypy” as a construct reflected in a sub-clinical mental and behavioral state, manifesting itself through several phenotypic levels, such as the liability of schizotypal personality (27, 28). Although Schizotypy is still lacking a unified definition in literature, it helps to link a broad continuum of clinical and non-clinical manifestations to form a predictive factor of the probability of converging into psychosis (29–34).

Schizotypal personality disorder (SPD) involves cognitive, perceptual, and social affective processing distortions and impairment, and neural abnormalities and includes three dimensions: positive (e.g., delusions, hallucinations, paranoia, and magical beliefs), negative (anhedonia, alogia, affective flattening, avolition, and social withdrawal), and disorganization dimensions (eccentric behavior and odd thoughts) (33, 35–38). The three dimensions show stability across cultures (39).

Schizotypy, as well as psychopathy, psychosis, and borderline personality disorder, are associated with other factors, such as impaired mentalization [the reflective function (RF)] (40–44). “The mentalizing difficulties may constitute an important clinical assessment and early prevention treatment targets in adolescents who demonstrate schizotypal features” (45).

Additionally, mental impairment, emotional and behavioral changes, and a higher rate of chronic disease are associated with the traumatic life event and post-traumatic stress disorder (PTSD) (46–49). Regarding the COVID-19 outbreak, as a traumatic life event, including the long-home stay and quarantine, numerous severe disorders are reported like the fear of death (self and beloved), stigma, isolation, anxiety, insomnia, feelings of helplessness, the severe alteration of lifestyle and daily activity such as the absence of physical exercise and limitation or no recreational and occupational activities (14, 50–61). People who worked from home reported positive feelings (50, 62). People (including patients) able to work and perform occupational, productive, and structured activities in general (e.g., household chores, recreational activities, and physical exercise), especially during quarantine, are associated with lowering the levels of anxiety, depression, stress, and other disorders (14–16, 26, 63–69). The “activity level in individuals at risk of schizophrenia is extremely important from a preventive point of view” (69). Therefore, the goal-directed structured activity and motivation, in interaction with culture and personality, are crucial for executive function, self-regulation, and quality of life, all of which are factors that face impairment and deterioration in schizophrenia (70–75). In general terms, it is hypothesized that adolescents' mental health maybe is linked to the changes in the patterns of time usage in the last 20 years (76).

Furthermore, Cultural-historical activity theory (CHAT) “emphasizes the constant flow of activity as the source of mind and self” [(77), p. 484]. The activity is a *molar*, and its internalization forms the Dynamic System of Meaning (DSM) as a structure of the mind, where experience is synthesized, including action, desires, needs, goals, sensory inputs, and emotions. DSM mediates the interaction with the environment—from straightforward motor action to higher mental functions (71, 72, 78).

Thus, investigating the activity system, including goals, motivation, and the actual activity, is essential for this study.

## Materials and methods

Following China's COVID-19 Zero-Tolerance policy (the Zero-cases policy in China about COVID-19), which includes a range of measures, from partial to complete lockdown, including house/residence and medical centers quarantines, the study was conducted between the 10th of November 2021 and the 29th of March, 2022. During that period, Zhengzhou, the capital city of Henan province, where our study was conducted, was coming out from the flood that hit the city in July 2021. Due to the flood, more than 376 thousand people were relocated, and the daily life of millions was directly affected after the flood in terms of transportation, residency, workplaces, and food and water safety. In addition to the flood, Zhengzhou witnessed another COVID-19 outbreak resulting in a lockdown between July and August. Furthermore, in the course of data collection, the city (including Henan province) faced another outbreak in January 2022, leading to an increase in lockdown and medical testing conditions, imposing further travel bans and restrictions on citizens' movement and travelers' quarantine. Within the same period (January and February), China celebrated the Lunar new year (Spring Festival holidays), which is a time in which most students (who form the target of the current study) moved back to their hometowns in the province, further increasing public anxiety (7).

This study investigates the relationships between schizotypal traits, mentalization, stress, and the activity system (including goals and motivation) during the pandemic-related lifestyle.

It is hypothesized that mentalization, PTSD, the activity system's components, and schizotypy are associated. Individuals with an optimal level of mentalization, the existence of activities goals, and higher motivation will present a lower range of schizotypal traits, regardless of the level of post-traumatic stress.

Following this reasoning and, to grasp the complexity of the variables, a mixed-method is used, including open-ended questions to inquire about some of the activity system variables (e.g., activity flow, goals) and questionnaires regarding other variables (Schizotypal personality, mentalization, stress, and motivation). Moreover, focusing on a specific group of participants would be suitable for generating an in-depth understanding of the phenomenon (see the section below).

### Participants

Our participants are college students currently enrolled in Zhengzhou Normal University (ZZNU), Henan, China. Previous studies have noted that college students showed considerable mental health impact during the pandemic. For instance, a study by Ma et al. (54) on a large sample of college students (746,217 participants) from 180 Chinese colleges revealed that about 45% of the participants had mental health problems, including acute stress, depression, and anxiety symptoms. Another longitudinal study by Wang et al. (16) showed that the respondents in the age group from 12 to 21 years old in the second survey had a higher psychological impact than in the first survey due to prolonged lockdown because this group is comprised of students “who were affected by prolonged school closure, requiring online education support, and uncertainty about examinations and matriculation arrangements” and young students were identified as a target group “prone for the psychological impact of the current COVID-19 outbreak” [(16), p. 47]. Additionally, Wang et al. (15) noted that being a student is significantly associated with higher levels of depression, anxiety, stress, and psychological impact due to the outbreak. Other studies also noted that the psychological consequences of the COVID-19 epidemic on Chinese college students could be severe, including PTSD, stress, anxiety, and depression (79–81). Overall, adolescents may experience increased psychiatric disorders resulting from the COVID-19 pandemic, and they may be less tolerant to lockdowns (53, 82).

By targeting college students, this study goes along with the National and Henan province multiple policies to address mental health and develop a monitoring system for adolescents. This study is part of the “Ministry of Science and Technology of the People's Republic of China, National Foreign Expert Program ‘One Belt One Road' Innovative Talent Team regarding the development and application of intelligent monitoring and early warning system for adolescents psychological crisis.”

A total of 909 college students participated in the study. All the students were invited to voluntarily participate in the survey through the platform named *Wenjuanxing via Wechat* (the Chinese communication application) and using internet browsers. Participants were asked to read the instructions about the purpose and methods to fill out the questionnaire carefully. Participants were also informed that the survey was anonymous. Twenty-three students (2.5%) refused to participate in the study. Another 34 (3.7%) were excluded due to missing data or completing the questionnaires in a short time of fewer than 3 min (taking into consideration that the mean of total time spent on the questionnaires was 10 min), leaving 852 (93.7%) included in the analysis.

The sample size was derived, on one hand, following the feasibility in time limitation and accessibility in collecting the data that fulfill the measurement of the study's variables (related to the effect of quarantine and pandemic life conditions). In order not to take a long time to measure the variables after the outbreaks' period (the first one was in July–August 2021, and the second outbreak was in January 2022), and to stay within the period of winter vacation when the “allocation” of students-related conditions (traveling chance and requirements) take place between the University and their home-towns, the collection of data needed to be completed (in late March) so that the whole sample belong relatively to the same context.

On the other hand, from a statistical position, our sample size exceeds the required minimum sample size for three types of tests we are going to conduct in this study (Pearson correlation, One-way Anova, and multiple linear regression). Using the G^*^Power program (Version 3.1.9.6), for multiple linear regression, the required minimum sample size is 226, with a low effect size (0.15), confidence level [error probability (α)] is equal to 0.5, the power is equal to 0.80, and for 20 predictors. Also, for One-way Anova, the required minimum sample size is 266 with an effect size equal to 0.2294 (determined by G^*^Power) and confidence level is 0.05 and the power is equal to 0.80 when comparing 7 groups (that is the highest number of groups for one of our variables, i.e., the “Marital status” variable). In addition, for Pearson's correlation test, the required minimum sample size is 82, for medium effect size (0.3), the confidence level [error probability (α)] is equal to 0.05, and the power of 0.80.

Furthermore, our sample exceeds the sample size that satisfies the rule of thumb of 10 samples per every measurement variable suggested by Hair et al. (83). Since we have 21 main variables to be investigated, we need at least 210 participants (our samples exceeded that number). Furthermore, the population of ZZNU is around 16,500 students, and following the *Simple and random sampling scheme*, our sample exceeds the statistical *ideal* sample size, in this case, is around 376 participants for a confidence level of 95% and confidence interval (±) 5% (84). Also, our sample size exceeds the desired sample size for correlational (82 participants) and causal (64 participants) designs, as well as for interview-based studies averaged of 104 participants (raging from 2–720 interviewees) (85, 86).

In addition, in line with existing research, our sample size exceeds previous studies' sample sizes. For instance, the sample size in a study by Lincoln et al. (87) to measure the MMS in a non-clinical sample was 76 participants, and 575 participants from workers and students of one university in a study by Kemp et al. (88). Also, our study's sample exceeds the sample size (105 participants) in a study by Salaminios et al. (45) investigating the association between schizotypal personality features and mentalization and exceeds the sample size of another study (89) investigating psychological wellbeing of hospital medical staff (668 participants).

### Tools

#### Schizoptypal personality

Measuring the Schizotypal symptoms in non-clinical samples can be done through several psychometric tools, using self-report surveys/questionnaires as the most common in the field, such as the Schizotypal Traits Questionnaire (STQ), Wisconsin Schizotypy Scales (WSS), Schizotypal Traits Scale (STA), Oxford-Liverpool Inventory of Feelings and Experiences (O-LIFE), Schizotypal Personality Questionnaire (SPQ), and Schizotypal Ambivalence Scale (SAS), among others (90). However, this study will employ the Multidimensional Schizotypy Scale-Brief (MSS-B) (91) as a brief version of MSS that measures the three directions (sub-scales) of Schizotypy: Positive, Negative, and Disorganized (92). The brief version (MSS-B) adopts 38 items from the 77 items forming the original scale. MSS-B showed valid psychometric properties, and the three analogous subscales of the MSS and MSS-B tap comparable constructs demonstrated by Kemp et al. (93), with high concordance across separate testings. In addition, MSS-B has a good construct of validity (94). Furthermore, studies revealed that MSS is valid in the Chinese context (Positive Schizotypy dimension's Cronbach's α = 0.892, Negative Schizotypy dimension's Cronbach's α = 0.778, and Disorganized Schizotypy dimension's Cronbach's α = 0.896) [e.g., see (34, 38)].

For this study, the Chinese version was obtained from Professor Kwapil after personal communication *via* email.

#### Mentalization

Although there are several instruments for assessing mentalizing (95), few instruments have been developed with the goal of employment in quantitative large-scale studies (96). This study will adopt the Reflective Function Questionnaire (RFQ). RFQ is a 46-item self-report measure (41), that has been adapted to measure the reflective function of adolescents and young through the Reflective Function Questionnaire for Youth (RFQY) (44). Moreover, both RFQ and RFQY's Chinese versions showed good reliability and validity in the Chinese context (Cronbach's α = 0.747) (97, 98).

#### Stress

Post-Traumatic Stress will be measured by the Impact Event Scale-Revised (IES-R) (99). IES-R was originally developed by Horowitz et al. (100) and further developed by Weiss (101) to assess intrusive experience and avoidant behavior as an aspect of post-traumatic stress disorder. IES-R is comprised of 22 items forming three subscales: intrusion (8 items), Avoidance (8 items), and Hyperarousal (6 items). IES-R has convergent validity with diagnosed PTSD (102) and sound psychometric properties (101). Also, the Chinese version of IES-R (CIES-R) has shown valid and satisfactory psychometric properties in the Chinese context [Intrusion (Cronbach's α = 0.89), Avoidance (Cronbach's α = 0.85), and Hyperarousal (Cronbach's α = 0.83)] (103, 104). Furthermore, IES-R has been used in numerous studies to measure the psychological impact of COVID-19-related experiences [e.g., (15, 16, 54, 59, 60, 68, 105, 106)].

#### Activity systems components

As noted earlier, the activity system highly depends on the goal of the activity, the motivation, and the flow of the activity. Personal activities, goals, and motivation are measured through self-report questions (structured, open, and close-ended).

#### Activity type

The questions regarding the type of activity in which the participants engaged were based on the Time Use Survey (TUS) regarding the time use and activities (107)—a Shortened version of TUS employed in Hodgekins et al. (65)—and the Quarantine activity checklist (68). Participants were asked to define what activities they are into and the daily scale in which they practiced each activity (hours per day).

Furthermore, separate questions were introduced to inquire about participants' engagement in the jobs. It is another factor to define how much an individual is engaged in social life and attached to a structured activity. The questions ask whether the participants are working during their study, the type of work (a full/part-time job), their position (self-employed or salaried employee), and the last time they worked.

#### Activity flow

As described earlier in the introduction, the activity flow is considered crucial for mental consistency. Therefore, participants had to describe how the COVID-19 situation affected their activities.

#### Goals and motivation

Regarding *goals*, the participants were asked whether they have any goals that direct their life and the type of these goals. The goals were described in answers to open questions. Later in the analysis of the answers (see Table 1), we will observe different categories of goals, including short term, long term, tangible and abstract goals. Regarding *motivation*, the participants were asked to state the motivation level (8 levels ranging from *None* to *High*) and describe the type of motivation through four states (intrinsic, identified regulation, extrinsic, and amotivated). Four themes are used to represent these four states: to *have fun* and *feel comfortable* (intrinsic). To have *great interest to me* (for identified regulation). *I have no choice* (for extrinsic), and *No motivation and no good reason to pursue it* (for amotivated) (see Table 1). The questions about motivation were formulated following the Situational Motivation Scale (SIMS) (108).

**Table 1:** Goal existence, type, and term.

**Variable**	**(*n* = 852)**	**%**
**Goal exists**		
Yes	573	67.3
No	279	32.7
**Goal term**	**(*****n*** = **564)**	**%**
Short	6	1.06
Medium	292	51.77
Long	266	47.16
**Goal type**	**(*****n*** = **564)**	**%**
Abstract	124	21.99
Tangible	440	78.01
**Motivation level**	**(*****n***=**573)**	**%**
1	1	0.17
2	6	1.04
3	40	6.98
4	102	17.71
5	190	33.16
6	104	18.15
7	130	22.69
**Motivation source**	**(*****n*** = **573)**	**%**
Intrinsic (It's fun and makes me feel comfortable)	173	30.19
Identified regulation (It can be of great benefit to me)	354	61.78
Extrinsic (I have no choice; this is what I should do)	33	5.76
Amotivated (Although I have this purpose in life, I have no motivation and see no good reason to pursue it)	13	2.27

### Statistical analysis

#### Types of collected data (quantitative/qualitative)

Two types of data were collected: Quantitative data and qualitative data, collected through demographic questions (gender, age, whether the participant is the only child in the family, education, home town, marital status, having children, number of children) MMS-B, RFQY, IES-R, the questions about motivation level and source, activities that the participants are part in, time spent on activities, the existence of goals, is the activity affected by the pandemic, are the participants working or not, the type/position of their job, and the time since they left their job (how many years), are all quantitative. Regarding the activities the participants are part of, eleven activities were included in the survey. In addition, the participants were required to answer about their engagement in these activities. The eleven activities are (1) Research, (2) Sports, (3) Online gaming, (4) Taking care of family and/or children, (5) Volunteering, (6) Reading, and (7) Music and art events, (8) Shopping, (9) Housework, (10) Handicraft, and (11) Other.

However, the data obtained from the questions regarding how the pandemic affected the activities, and the types of goals are qualitative.

#### Interpreting and analyzing qualitative data

Regarding the quantification of qualitative answers, the answers were organized under general themes and then coded for data manipulation.

Regarding the question about *how the pandemic affected the activity*, the participants' answers were organized under nine general themes (coded from 1 to 9): (1) shopping (when the participants explicitly declare that their shopping habits are affected either by the decrease of the outdoor shopping or the increase of the online shopping), (2) the activity in general decreased, (3) causing the participants to feel upset (when the participants expressed a feeling and a position toward the changes in their activity such as the loss of motivation, the influence on life, a bad mood, the inconveniency of the situation and the feeling of decrease in freedom), (4) the inability to go out and travel, (5) doing sports is affected, (6) the total halt of activity, (7) the education is affected, (8) more time spent on some aspects like playing online games, reading, and listening to music, (9) and the final theme is the effect on financial status (since part-time jobs were stopped).

Regarding the quantification of goals type (abstract and tangible), the answers that hold the aspects of social contributions and moral values (e.g., becoming a good/excellent teacher, taking care, and supporting their own family and friends), self-development (e.g., when participants declare that the graduation has a great impact on him/her; the goal to increase knowledge; to live a happy life; changing lifestyle), and other goals related to art, music, and becoming a writer/novelist, are considered as *abstract goals*. However, the goals directed toward direct desires such as educational (e.g., pass the exam, which formed the majority of the answers; learn English), financial (earn more money), career (find a job; to note here that we differentiated between the desire to have a job and the desire to become a good teacher since the first does not hold an explicit moral aspect), ownership and consumption (buying fancy cars and houses), and fitness-related (lose weight and perform the physical activity).

For the goal term, as noted earlier, we came to classify the answers according to three themes and coded them as such [(1) short, (2) medium, and (3) long terms]. The short-term goals were mainly based on answers such as: to study, or to study well, and to attend the classes, which refer to an ongoing process with a lack of futuristic tendency. However, taking into consideration them being college students, the participants' goals to graduate/pass the exam and other studies-related are classified as medium-term, while the goals to find a job or to reach certain achievements (publish a novel, contribute to teaching carrier, support the family, earning considerable money, and buying houses and cars, to move to other cities) are considered as long-term goals.

Regarding the *credibility* and *reliability* of the collected data, since the data were collected online, the responses are the participants' own words, so neither a moderator nor the researchers themselves engaged in recording or writing the responses. The collected answers are in the voice of the participants, so the researchers' *bias* is not affecting the obtained data and their *neutrality* is supported. In addition, two sorts of triangulations are used. First, is it data triangulation in terms of time is used to ensure multiple sources of data. The data were collected in two phases, the first was between the first of November and December 2021, and the second was March 2022. The second is the investigators' triangulation. For the coding and categorization of answers, multiple researchers have triangulated their classifications of answer themes.

To support the coding of the answers, previous resources are followed. For instance, regarding the classification of goals types (tangible/concrete and abstract) and range (short, medium, long), in the framework of control theory, Emmons (109) investigated the level of generality of personal goals in relation to mental illness and wellbeing, and they proposed two extremes. First are the broad goals. Here a person is called high-level strivers (their projects are more meaningful), and the implication/purpose of the goal is important, e.g., trying to make others happy, increase my knowledge, and be fun (in our case, we obtained answers such as *to improve myself*; see Table 2). The second extreme is the superficial one. Here a person is called *a low-level striver* (their project is molecular), and the way the actions are carried out is more important, e.g., looking physically fit, and buying new luggage (in our case, we obtained answers such as *buying houses/cars*; see Table 2). The collected data are represented in tables and analyzed with statistical tests, being as comprehensive and inclusive with quantitative aspects, as another factor of the study's *reliability*.

**Table 2:** Goals type and range examples.

**Goal' type and range**	**Participants' answers**
1. Tangible (Short-term)	Study
2. Tangible (Short-term)	To attend the classes
3. Tangible (Medium-term)	Postgraduate entrance examination
4. Tangible (Medium-term)	To find a job
5. Tangible (Medium-term)	To graduate
6. Tangible (Medium-term)	To pass English language tests (IELTS)
7. Tangible (Medium-term)	Lose weight
8. Tangible (long-term)	Travel
9. Tangible (long term)	Make Money
10. Tangible (long term)	To settle in a big city
11. Tangible (long term)	Buy a house
12. Tangible (long term)	Buy a fancy car
13. Abstract (Medium-term)	Read a few books to improve yourself
14. Abstract (Long-term)	Enrich my self
15. Abstract (Long-term)	To share responsibilities with the family
16. Abstract (Long-term)	Become a person with developed morality and intelligence
17. Abstract (Long-term)	To engage in music
18. Abstract (Long-term)	To be an excellent teacher
19. Abstract (Long-term)	Improve my life quality
20. Abstract (Long-term)	To reach financial independence
21. Abstract (Long-term)	To write my own novel

As another factor in evaluating *credibility, familiarity* with the context is ensured because some of this study researchers are natives and belong to the setting where the study was conducted while others (who are not natives) had been living in China, and in the city of Zhengzhou for several years. Therefore, they are familiar with the conditions and cultural tendencies surrounding the study, as well as the changes in these conditions in the recent period. Being grounded in the set brings the researchers closer to the study's setting, hence, it reduces the level of preconceptions and misassumptions and increases their reflexivity. Therefore, *reflexivity*, in addition to the description of the context (regarding the *transferability* of the study) in the introduction of this section (*Materials and tools*) and reporting the sampling method, along with *credibility*, are other factors of *validity* and trustworthiness of the study [e.g., see, (110, 111)].

#### Types of variables (numerical/categorical)

Regarding the studies' variables, the MMS-B (for measuring Schizotypal personality) (higher score on each subscale means a higher level of Schizotypy), RFQY (for measuring mentalization, when a higher score means higher mentalization level), and IES-R (for measuring stress, where a higher score means higher PTSD, with the cutoff is equal to 24 so the participant's PTSD level is a clinical concern), provide a total score, so when conducting tests they all represent numerical data. Also, the age, whether the participant is the only child in the family, marital status, number of children, the duration of leaving work (in years), time spent on each activity (hours per day), and the level of motivation (ranges from 0 to 7) both represent the numerical type of data.

However, some demographic data (gender, education, whether the participant is the only child in the family, home town, marital status, having children) the existence of goals (yes/no), goal types (abstract/tangible; and short, medium, or long term), motivation source (intrinsic, identified regulation, extrinsic, amotivated), whether the activity is affected by the pandemic-related life conditions (yes/no), whether the participant worked or working (yes/no), the type of their job (full- time or part-time) and their job position (self-employed or salaried employee), are all categorical data. All categorical data will be coded numerically to be investigated.

#### Statistical tests

Using SPSS-IBM (22.0), correlation and regression tests were conducted to investigate the relationships among variables.

Pearson correlation (Two-tailed) is used to investigate the associations between continuous variables (e.g., PTSD, mentalization, Schizotypy, activities' total time, Motivation level), while the One-Way ANOVA test (with Tukey's *post-hoc* test and a significance level of 0.05) is used to investigate the differences among numerical and categorical variables, and two, among categorical variables. The dependent variables are the Schizotypal dimensions, PTSD, and reflective function, and the independent variables are the demographic variables, and the activity system's variables (goals, motivation, activity time, and activity type).

Moreover, Multiple linear regression (with a 95% confidence percentage) was used to investigate predictive relationships among variables. The dependent variables are Positive Schizotypy, Negative Schizotypy, and Disorganized Schizotypy, while the independent variables (predictors) for each one of the dependent variables is: the demographic variables (e.g., gender, age, being the only child in the family, education level), PTSD, reflective function level, and activity system-related variables (e.g., goals, motivation, activity time, and activity type).

## Results

### Descriptive statistics

#### Demographic

Demographic characteristics of the sample were: Mage = 20.06 years, SD = 1.45, range 18–28 years. The participants' age satisfies the conditions of RFQY and MMS. RFQY targets both adolescents and young (44), and MMS-B targets a sample between 18 and 60 years old (91). Ninety-one percent (91%) were female.

Regarding the gender imbalance in the sample, commonly, the majority of the Normal Universities students in China are females. In our case, the male/female ratio in the population of ZZNU is 1:8 (87.5% are females), which is nearly similar to the percentage in our sample. Therefore, the original skewness is in the population. In the case of an imbalanced sample, it is important to check whether the sample is representative, and what is more important is to compute the power of interaction [e.g., see, (112)]. Therefore, by conducting a *post-hoc* test, with G^*^Power, to calculate the power of gender interaction (with other variables) for ANOVA (our sample size represented power of 0.7 when the effect size is 0.252, the level of confidence is 0.05, and the number of groups is 835 (the number of groups is the multiplication of the number of levels forming the independent variables that gender may interact with, e.g. the one *child* variable has 2 levels, *education* has 5 levels, *the home town* has 2, *goal range* has 2, *goal type* has 3, *motivation source* has 4) and the numerator is 35 [this is the result of multiplying each variable's number of levels minus one, i.e., (variable **one**' number of levels-−1)^*^ (variable **two**' number of levels-−1)^*^ variable **three**' number of levels-−1….]; which means that even if gender is interacting with other independent variables at the same time, this study's sample size supports strong power. There is a 70% chance of correctly rejecting the null hypothesis of no difference with a total of 852 participants.

For other demographic data about being the only child, education, hometown, marital status, and having children or not, please see Table 3.

**Table 3:** Demographic characteristics of participants.

	**Mean**	**%**	**SD**	**Range**
Age	20.06		1.45	18–28
	***N***		**%**	
**Gender**			
Female	775	91	91	
Male	77	9	9	
**Marital Status**			
Single	639		75	
Married	1		0.1	
Widow	1		0.1	
In a relationship	196		23	
Other	15		1.8	
**Number of children**			
One	125		14.7	
More	727		85.3	
**Hometown**	***N***		**SD**	
City	202		23.7	
Town	154		18.4	
Countryside	496		58.2	
**Education**			
Bellow Junior college	2		0.2	
Junior college	123		14.4	
Undergraduate	724		85	
Master's	2		0.2	
Ph.D.	1		0.1	

#### Activity related variables

Overall, the activities engagement time (hour/day) Mean = 9.96 (SD= 9.41) (see Table 4 for more about activity and working history). About 42% considered that their activities were affected by pandemic-related conditions. By analyzing participants' answers, we finally identified nine general categories. About 33.81% of them stated that pandemic-related conditions reduced their activity time, 22.16% stated that they were not able to go out and travel, and 15.63% considered that their shopping habits were affected or stopped. In addition, 12.5% considered that their activity is stopped. The answers of 3.53% were related to sports activities. A total of 3.98% expressed that their mood was affected. However, 2.84% considered that, during closure time, they had more time for certain activities such as reading, online games, and staying with the family. And only 1.7% answered educational-related effects (see Table 5). Examples of how the pandemic affected the participants' activities are included in Table 6.

**Table 4:** Activity type and working job.

	**(*n* = 852)**	**%**
**Activity type**		
Research	30	3.5
Sports	353	41.4
Online games	378	44.4
Take care of children and family members	134	15.7
Volunteer work	175	20.5
Reading	522	61.3
Music and art events	424	49.8
Shopping	596	70.00
Housework	370	43.4
Handicraft	226	26.5
Other	216	25.4
**Activity time (Hour per day)**	**Mean**	**SD**
	9.96	9.42
**Working job experience**	**(n = 852)**	**%**
**Working now**		
Yes	69	8.1
No	783	91.9
	**(*****n*** = **69)**	**%**
**Working time**		
Full-Time	3	4.35
Part-Time	66	95.65
**Job type**		
Self-employed	5	7.25
Salaried employees	64	92.75
	**(*****n*** = **852)**	**%**
**Worked before**		
Yes	180	21.1
No	652	76.5
No answer	20	2.3
**Year of stopping work**	**(*****n*** = **180)**	**%**
2012	1	0.56
2017	1	0.56
2018	4	2.22
2019	9	5
2020	23	12.78
2021	95	52.78
2022	25	13.9
No answer	22	12.2
**Job type**		
Self employed	15	8.3
Salaried employees	165	91.7

**Table 5:** How the activity is affected by the pandemic.

**Main categories**	***N = 352***	**%**
Activity is reduced	119	33.81
Unable to go out/travel	78	22.16
Shopping affected	55	15.63
Activity is stopped	44	12.5
Sports is affected	23	6.53
Mood is affected	14	3.98
More time for certain activities (reading, online games, staying with family…)	10	2.84
Education is affected	6	1.7
Financial related	3	0.85

**Table 6:** Examples of how participants' activities are affected.

**How activity affected**	**Participants' answers**
1	My activity is reduced
2	Can't realize my plans
3	Stop going to the gym
4	Unable to go out
5	Can't travel
6	Can't shop, only online shopping
7	Can't buy the materials you want
8	Inconvenient situation
9	Learning more online games and shopping is reduced
10	Increase online and indoor activity
11	Lost motivation
12	Can't be with relatives and friends
13	The mood is affected
14	Very uncomfortable
15	School's schedule is compressed and learning tasks are tense
16	More time for reading
17	More time for handicraft
18	More time with family
19	More time for online games

Furthermore, 67.3% reported that they have plans and goals directing their activities. Regarding activities type, goals type and range, motivations type and source, and working status, see Table 1. Examples of goals types are presented in Table 2.

#### IES-R, RFQY, and MMS-B

The sample showed a Mean of 22.2 (SD = 14.9) on IES-R (stress level) which is considered lower than the limit where PTSD becomes a clinical concern (54% of participants expressed a low level below 24). A total of 22.2% belonged to the level where PTSD is a clinical concern, 5.8% of participants had a probable diagnosis of PTSD, while 18% expressed high PTSD. For IES-R subscales, Hyperarousal's Mean is equal to 0.88 (SD = 0.72); Avoidance's Mean is equal to 1.08 (SD = 0.75), and Intrusion's Mean is equal to 1.11 (SD = 0.71). The mean reflective function score as determined by the RFQY was 5.71 (SD = 0.43) with a minimum of 3.39 and a maximum of 6.48.

Around 46% of the participants had stress levels above the cut-off (score = 24 on IES-R), with a Mean score = 22.9 (SD = 14.9) (see Table 7), which goes with previous studies that also used IES-R to measure stress levels in the Chinese context during the pandemic. For Chen et al. (106) and Ting et al. (113) the considerable stress percentage was 44.5% for sample's *N* = 9,225 (Median is provided = 16.0) and 45.2% (Mean score = 26.61; SD = 17.95), respectively. However, our sample's PTSD mean was lower than that of studies conducted in 2020 [e.g., see, (15)], which may consistent with a longitudinal study by Wang et al. (16) that showed that the PTSD Mean score decreased over time [from 32.98 (SD = 15.42) to 30.76 (SD = 15.42)]. Also, along with previous studies, our results did not show any difference in age, marital status, and employment record on the PTSD score (15, 113). However, regarding gender difference in PTSD score, our study showed that males reported significantly higher levels than females, which contrasts with previous studies (15, 16, 54) (see Table 8). Also, unlike Ma's et al. (54) outcomes, different educational levels did not show any significant difference in PTSD levels.

**Table 7:** IES-R, RFQY, and MMS-B descriptive results.

**IES-R**		**(*n* = 852)**	**Minimum**	**Maximum**
**Variable**				
IES-R total	*M*	22.9	0.00	88.00
	*SD*	14.9		
	***n***	**%**		
IES-R (>24)	460	54		
IES-R (≥24)	189	22.2		
IES-R (≥33)	49	5.8		
IES-R (≥37)	154	18		
**IES-R subscale**		**(*****n*** = **852)**		
Avoidance	*M*	1.08		
	*SD*	0.75		
Intrusion	*M*	1.11		
	*SD*	0.71		
Hyperarousal	*M*	0.88		
	*SD*	0.72		
**RFQY**				
RFQY total	*M*	5.71	3.39	6.48
	*SD*	0.43		
**MMS-B subscales**				
Negative schizotypy	*M*	3.99	0.00	13.00
	*SD*	2.52		
Positive schizotypy	*M*	2.41	0.00	13.00
	*SD*	2.84		
Disorganized schizotypy	*M*	1.87	0.00	12.00
	*SD*	2.64		

**Table 8:** Means of significant variables in ANOVA.

**Variables**	***N***	***Mean***	***SD***
Pandemic Effect*Positive schizotypy			
Yes	358	2.6872	3.01
No	494	2.2105	2.67
Pandemic Effect*PTSD			
Yes	358	25.0503	15.621
No	494	21.3239	14.253
Goals existence*Pandemic Effect			
Yes	357	0.73	0.443
No	494	0.63	0.483
Goals existence*Disorganized Schizotypy			
Yes	573	1.644	2.393
No	279	2.319	3.047
Goals existence*Reflective function			
Yes	573	5.747	0.377
No	279	5.640	0.503
Goals' range*Negative schizotypy			
Short-Term	6	7.333	4.320
Medium-Term	292	4.007	2.204
Long-Term	266	3.748	2.360
Goals' range*Disorganized schizotypy			
Short-Term	6	4.500	4.324
Medium-Term	292	1.527	2.111
Long-Term	266	1.748	2.615
Goals' range*Reflective function			
Short-Term	6	5.367	0.787
Medium-Term	292	5.753	0.327
Long-Term	266	5.747	0.413
Goals' type*Negative schizotypy			
Tangible	440	4.082	2.383
Abstract	124	3.347	2.052
Goals' type*Disorganized schizotypy			
Tangible	440	1.693	2.395
Abstract	124	1.557	2.451
Goals' type*Reflective function			
Tangible	440	5.743	0.352
Abstract	124	5.757	0.457
Gender*Negative schizotypy			
Female	775	3.903	2.424
Male	77	4.844	3.257
Gender*Positive schizotypy			
Female	775	2.294	2.721
Male	77	3.584	3.676
Gender*Disorganized schizotypy			
Female	775	1.743	2.512
Male	77	3.091	3.499
Gender*PTSD			
Female	775	22.361	14.377
Male	77	28.208	19.117
Gender*Reflective function			
Female	775	5.723	0.414
Male	77	5.596	0.515
Education*Negative schizotypy			
Bellow junior college	2	5.500	0.707
Junior college	123	3.431	2.875
Undergraduate	724	4.087	2.445
Master	2	0.00	0.00
PhD	1	6.00	0.00
Children number*Positive schizotypy			
One	125	3.200	3.429
More	727	2.275	2.708
Gender*Shopping			
Male	77	0.42	0.496
Female	775	0.73	0.445
Gender*Children number			
Male	77	1.73	0.448
Female	775	1.87	0.341
Gender*Online games			
Male	77	0.82	0.388
Female	775	0.41	0.491

For schizotypal personality as measured by MMS-B, the Negative Schizotypy's Means is equal to 3.99 (SD = 2.52), Positive Schizotypy's Mean is 2.41 (SD = 2.84), and the Disorganized Schizotypy's Mean is 1.87 (SD = 2.64) (see Table 7). Regarding schizotypal traits, our results showed lower levels on all the MSS subscales comparing previous studies in the Chinese context and higher than other studies in other contexts. Our scores were 3.99, 2.41, and 1.87, showing lower levels compared to 6.73, 3.54, and 3.63 in Wang et al. (38), and showing higher levels compared to 1.55, 2.03, and 1.81 in Gross et al. (91) (among college students in the U.S.), and compared to 0.93, 1.10, and 1.15 in Lincoln et al. (87) (among university students in the U.S.), and compared to 1.73, 1.30, and 1.56 in Kemp et al. (88), on Negative, Positive, and Disorganized subscales, respectively.

### Means difference, correlation, and linear regression

Using SPSS-IBM (22.0) results partially supported previous studies' results as well as showed the association between activity system and Schizotypal traits.

Comparing means by performing one-way ANOVA revealed a statistically significant difference between the participants who reported the effect of the pandemic on their activity in terms of Positive Schizotypy and PTSD levels [*F*_(1, 850)_ = (5.871), *p* = 0.016; *F*_(1, 850)_ = (13.082), *p* < 0.001, respectively]. Participants who reported that their activities were affected showed higher levels of Positive Schizotypy and PTSD (see Tables 8, 9).

**Table 9:** One-way ANOVA.

**Variables**	***df***	***F***	***p***
Pandemic effect*Positive schizotypy			
Source			
Between groups	1	5.871	0.016
Within groups	850		
Total	851		
Pandemic Effect*PTSD			
Source			
Between groups	1	13.082	0.000
Within groups	850		
Total	851		
Goals existence*Pandemic effect			
Yes	357	10.358	0.001
No	494		
Goals existence*Disorganized schizotypy			
Source			
Between groups	1	12.410	0.000
Within groups	850		
Total	851		
Goals existence*Reflective function			
Source			
Between groups	1	12.001	0.001
Within groups	850		
Total	851		
Goals' range*Negative schizotypy			
Source			
Between groups	3	4.652	0.003
Within groups	848		
Total	851		
Goals' range*Disorganized schizotypy			
Source			
Between groups	3	6.005	0.000
Within groups	848		
Total	851		
Goals' range*Reflective function			
Source			
Between groups	3	5.373	0.001
Within groups	848		
Total	851		
Goals' type*Negative schizotypy			
Source			
Between groups	2	4.755	0.009
Within groups	849		
Total	851		
Goals' type*Disorganized schizotypy			
Source			
Between groups	2	5.048	0.007
Within groups	849		
Total	851		
Goals' type*Reflective function			
Source			
Between groups	2	5.651	0.004
Within groups	849		
Total	851		
Gender*Negative schizotypy			
Source			
Between groups	1	9.845	0.002
Within groups	850		
Total	851		
Gender*Positive schizotypy			
Source			
Between groups	1	14.666	0.000
Within groups	850		
Total	851		
Gender*Disorganized schizotypy			
Source			
Between groups	1	18.597	0.000
Within groups	850		
Total	851		
Gender*PTSD			
Source			
Between groups	1	10.838	0.001
Within groups	850		
Total	851		
Gender*Reflective function			
Source			
Between groups	1	6.327	0.012
Within groups	850		
Total	851		
Education*Negative schizotypy			
Source			
Between groups	4	3.405	0.009
Within groups	847		
Total	851		
Children number*Positive schizotypy			
Source			
Between groups	1	11.434	0.001
Within groups	850		
Total	851		
Gender*Shopping			
Source			
Between groups	1	33.678	0.000
Within groups	850		
Total	851		
Gender*Children number			
Source			
Between groups	1	10.849	0.001
Within groups	850		
Total	851		
Gender*Online games			
Source			
Between groups	1	50.864	0.000
Within groups	850		
Total	851		

Also, results revealed a statistically significant difference between the participants who reported the existence of goals in terms of Disorganized Schizotypy level [*F*_(1, 850)_ = (12.410), *p* < 0.001] and reflective function [*F*_(1, 850)_ = (12.001), *p* = 0.001]. Participants with goals directing their activity reported lower Disorganized Schizotypy and higher reflective function (see Tables 8, 9). Also, having different goals' range was statistically significant in terms of Negative and Disorganized Schizotypy [*F*_(3, 848)_ = (4.652), *p* = 0.003; *F*_(3, 848)_ = (6.005), *p* < 0.001; respectively], and reflective function [*F*_(3, 848)_ = (5.373), *p* = 0.001]. Participants with medium-term goals reported the lowest Disorganized Schizotypy and the highest level of reflective function (the difference in Disorganized Schizotypy and reflective function levels between medium and long-term goals groups is small; see Tables 8, 9). Additionally, participants with long-term goals reported the lowest Negative Schizotypy. However, participants with short-term goals reported the highest Negative and Disorganized Schizotypy and lowest reflective function. Regarding the goals' type, there was statistically significant difference between participants with tangible and abstract on Negative and Disorganized Schizotypy [*F*_(2, 849)_ = (4.755), *p* = 0.009; *F*_(2, 849)_ = (5.048), *p* = 0.007, respectively], as well as on reflective function [*F*_(2, 849)_ = (5.651), *p* = 0.004]. Participants with abstract goals reported higher reflective function and lower levels of Negative and Disorganized Schizotypy (see Tables 8, 9). Examples of participants' answers regarding their goals are introduced in Table 2.

Results showed no significant difference among participants with different types of motivation sources.

After conducting the Pearson correlation test, results showed that the activities' total time (for all activities combined) was not correlated with Schizotypy, but was negatively correlated with reflective function and positively correlated with PTSD. However, research and volunteering were positively correlated with Positive Schizotypy, Disorganized Schizotypy, and PTSD, but negatively correlated with reflective function. Sports and shopping were negatively correlated with Negative Schizotypy and reflective function. While online games activity was positively correlated with Disorganized Schizotypy and PTSD. Moreover, music/art and handicraft activities are not correlated with any type of Schizotypy, but both were negatively correlated with reflective function, and music/art activity was positively correlated with PTSD. The reflective function was negatively correlated with both Positive and Disorganized Schizotypy, while PTSD (measured by IES-R) was positively correlated with three subscales of Schizotypy. Moreover, the three IES-R subscales (Intrusion, Hyperarousal, and Avoidance) were positively correlated with the three types of Schizotypy. However, the Motivation level was negatively correlated with Disorganized Schizotypy and positively correlated with reflective function (see Table 10).

**Table 10:** Correlations results.

**Variables**	**Negative MMS**	**Positive MMS**	**Disorganized MMS**	**RFQ-Y**	**PTSD**
1. Activity time	−0.054	0.033	0.032	−0.106**	0.086*
2. Act1 (Research activity)	0.026	0.088*	0.053	0.022	0.079*
3. Act2 (Sports)	−0.103**	−0.017	0.000	−0.077*	0.032
4. Act3 (Online games)	0.041	0.058	0.085*	−0.038	0.088*
5. Act5 (Volunteer)	0.035	0.076*	0.074*	−0.112**	0.107**
6. Act7 (Music/Art)	−0.029	0.038	0.020	−0.102*	0.071*
7. Act8 (Shopping)	−0.076*	−0.040	−0.034	−0.072*	0.033
8. Act10 (Handicraft)	−0.019	0.014	−0.012	−0.100*	−0.013
9. Act11 (Other)	−0.024	−0.071*	0.016	−0.008	−0.013
10. Motivation level	−0.050	−0.001	−0.114**	0.088*	−0.019
11. RFQY	−0.053	−0.117**	−0.215**	–	0.040
12. IES-R	0.283**	0.431**	0.410**	0.040	–
13. IES-Avoidance	0.259**	0.384**	0.360**	0.043	–
14. IES-Intrusion	0.254**	0.420**	0.379**	0.038	–
15. IES-Hyperarousal	0.286**	0.409**	0.424**	0.029	–

*p < 0.05, **p < 0.01.

Regarding demographic characteristics, comparing means by performing one-way ANOVA revealed a statistically significant difference between males and females regarding the three types of Schizotypy [*F*_(1, 850)_ = (9.845), *p* = 0.002, for Negative Schizotypy; *F*_(1, 850)_ = (14.666), *p* < 0.001, for Positive Schizotypy; and *F*_(1, 850)_ = (18.597), *p* < 0.001, for Disorganized Schizotypy]. Males has higher levels than females in the three types of Schizotypy. Also, there was a statistically significant difference between males and females regarding PTSD [*F*_(1, 850)_ = (10.838), *p* = 0.001] and reflective function [*F*_(1, 850)_ = (6.327), *p* = 0.012]. Males reported higher PTSD but lower reflective function. Moreover, males and females showed a significant difference in online games, shopping, and being the only-child in the family. Males had significantly higher scores on online game activity than females [*F*_(1, 850)_ = (50.864), *p* < 0.001], they belong to a one-child family more than females [*F*_(1, 850)_ = (10.849), *p* = 0.001], and they were less engaged in shopping activity than females [*F*_(1, 850)_ = (33.678), *p* < 0.001] (Tables 8, 9).

Regarding education, there was a statistically significant difference between educational levels regarding Negative Schizotypy [*F*_(4, 847)_ = (3.405), *p* = 0.009]. Even by excluding the Ph.D., Master, and Bellow Junior College participants because the Ph.D. level includes only one participant, while the Master's and Bellow Junior College level include only two participants, both Junior college and undergraduate students had significantly different Negative Schizotypy [*F*_(1, 845)_ = (7.176), *p* = 0.008]. Undergraduate students reported higher levels than students in Junior College. Also, there was a statistically significant difference between participants regarding the number of children in their family for Positive Schizotypy [*F*_(1, 850)_ = (11.434), *p* = 0.001] as well as for Disorganized Schizotypy [*F*_(1, 850)_ = (5.860), *p* = 0.016]. Being the only child in the family is associated with high Positive and Disorganized Schizotypy levels (see Tables 8, 9).

Other demographic variables (age, hometown, marital status, job engagement, and job history) did not show any significant difference among participants on reflective function, PTSD, and Schizotypy.

Moreover, to investigate variables predicting Schizotypy, multiple linear regression was used to test whether the activity system's variables (goal's type and range, motivation's level, and source, activity total time, if activity is affected by the pandemic), other correlated demographic variables (gender, the only child in the family), as well as reflective function and PTSD.

For Positive Schizotypy, the fitted regression model was: Positive Schizotypy = 7.849 −0.623^*^(gender)−0.645^*^(Only child)−0.110^*^(Activity affected by pandemic) + 0.080^*^(PTSD Level) −0.826^*^(Reflective function level). The overall regression was statistically significant [*R*^2^ = 0.216, *F*_(5, 846)_ = 46.571, *p* ≤ 0.001]. About 21.6% of data can be explained by the model which is moderate level. It was found that gender, only child, PTSD, and reflective function significantly predicted Positive Schizotypy (β = −0.063, *p* = 0.042; β = −0.080, *p* = 0.009; β = 0.423, *p* ≤ 0.001; β = −0.124, *p* ≤ 0.001; respectively) (see Table 11). The pandemic effect on activity was not found to be a significant predictor for Positive Schizotypy.

**Table 11:** Linear regression summary for positive schizotypy.

	**B**	**β**	**t**	***p***	**F**	**df**	**Sig**.	***R^2^***
(Constant)	7.849		5.879	0.000	46.571	5, 846	≤ 0.000	0.216
Gender	−0.623	−0.063	−2.033	0.042				
Only child	−0.645	−0.080	−2.216	0.009				
The effect of pandemic	−0.110	−0.019	−0.620	0.535				
PTSD	0.080	0.423	13.672	0.000				
Reflective function	−0.826	−0.124	−4.031	0.000				

For Negative Schizotypy, the model above was found to be less predictive. The fitted regression model was= 2.900–0.745^*^(gender) + 0.512^*^(Education) + 0.046^*^(PTSD). The overall regression was statistically significant [*R*^2^ = 0.091, *F*_(3, 848)_ = 28.434, *p* ≤ 0.001]. It is only 9.1% of data can be explained by the model which is relatively low level. It was found that gender, education, and PTSD significantly predicted Negative Schizotypy (β = −0.085, *p* = 0.011; β = 0.076, *p* = 0.021; β = 0.272, *p* ≤ 0.001, respectively) (see Table 12).

**Table 12:** Linear regression summary for negative schizotypy.

	**B**	**β**	**t**	***p***	**F**	**df**	**Sig**.	***R^2^***
(Constant)	2.900		2.909	0.004	28.434	3, 848	≤ 0.000	0.091
Gender	−0.745	−0.085	−2.557	0.011				
Education	0.512	0.076	2.306	0.021				
PTSD	0.046	0.272	8.239	0.000				

Finally, for Disorganized Schizotypy, the model above was found to be predictive (relatively less than Positive Schizotypy and higher than Negative Schizotypy). The fitted regression model was = 9.539–0.714^*^(gender) −0.429^*^(Goal existence) + 0.072^*^(PTSD) −1.343^*^(Reflective Function Level).

The overall regression was statistically significant [*R*^2^ = 0.234, *F*_(4, 847)_ = 64.756, *p* ≤ 0.001]. A total of 23.4% of data can be explained by the model which is a moderate level. It was found that gender, the existence of goals, PTSD, and reflective function significantly predicted Disorganized Schizotypy (β = −0.078, *p* = 0.011; β = −0.076, *p* = 0.012; β = 0.408, *p* ≤ 0.001; β = −0.216, *p* ≤ 0.001, respectively) (see Table 13).

**Table 13:** Linear regression summary for disorganized schizotypy.

	**B**	**β**	**t**	***p***	**F**	**df**	**Sig**.	***R^2^***
(Constant)	9.539		8.189	0.00	64.756	4, 847	≤ 0.000	0.234
Gender	−0.713	−0.078	−2.548	0.011				
The existence of goals	−0.429	−0.076	−2.514	0.012				
PTSD	0.072	0.408	13.445	0.000				
Reflective function	−1.343	−0.216	−7.101	0.000				

## Discussion

Results revealed that the main elements of the activity system (goal existence, goal type and terms, motivation, the activity flow in terms of being affected by the pandemic-related lifestyle), in addition to reflective function, are associated with schizotypal traits, regardless the level of PTSD.

Our results were similar to the previous studies about the association of three subscales of schizotypal traits with psychological trauma, affective symptoms, and stress [e.g., see (48, 88, 114–116)]. Furthermore, our results showed an association between reflective function and schizotypal traits (Positive and Disorganized schizotypy) in alignment with previous studies (40–45) (see Table 9). Also, as some studies in the Chinese context showed [e.g., see (117)], the male participants scored higher than females on schizotypal traits.

In addition, by considering the weight of social variables and social isolation as noted in previous studies [e.g., see (54, 113)], being not the only child in the family (more social connections and social support) is associated with a lower level of Positive Schizotypy. Also, being an undergraduate student is associated with high Negative Schizotypy (although both groups did not show any significant difference in terms of PTSD and reflective function) which goes partly with some previous studies where the higher the educational level we have, the higher some of the psychopathological aspects are (Depression, Stress, and Anxiety) [e.g., (16, 54, 68, 105, 118)].

Along with the goal-oriented aspect of the activity, and since goals play a crucial role in structuring and organizing both the mental and practical activities, our results showed that the existence of personal goals is associated with a lower level of Disorganized Schizotypy. The existence of a goal was one of the predictors of the regression model regarding the Disorganized Schizotypy (see Table 13). In addition, higher motivational levels appeared to be associated with lower levels of Disorganized Schizotypy. Also, our results showed the association of the goal's existence with a higher reflective function. And having abstract goals appeared to be associated with lower levels of Negative and Disorganized Schizotypy, and with higher reflective function.

For goal range, having Medium-term goals appeared to be associated with the lowest Disorganized Schizotypy and highest reflective function. Therefore, one may say that in the context where daily life is disturbed, the existence of goals, and having long-term and abstract goals, with a higher level of motivation is associated with less withdrawal represented by Negative schizotypal traits. Savla et al. (119) argued that “better abstraction was associated with… shorter illness duration, and functional capacity” [(119), p. 1]. It seems also that during this disturbed flow period, having Medium-term goals appeared to be more associated with keeping the mind structured and higher reflective function (compared to short and long-term goals). Overall, it seems that the existence of goals is associated with less Schizotypy, but the type and range of the goals are differently associated. Neither being *drawn* to the current moment nor *dreaming* about the future may be healthier since having a higher risk to be linked with a disorganized mind (although the difference between medium and long-term goals is not high in terms of Disorganized Schizotypy) (see Table 8).

Furthermore, in addition to goals and motivation, the real flow of the activity is also associated with schizotypal traits. The participants who reported that their activity was affected by the pandemic-related conditions scored higher on Positive Schizotypy. It seems that in the conditions where the activity is halted, the personality tends to mentally compensate for the halted desired goals and activities through the inflation of certain traits. Here, the schizotypal traits appear to be a reaction to the disturbance of the activity system (120). Being governed by a dynamic system of meanings, the mind seeks balance among the components of that system. In our case, the withdrawal from social life requires compensation which appears through the inflation of mind activities represented by Positive Schizotypal traits (e.g., delusions, hallucinations, paranoia, and magical beliefs). This is supported by the fact that the Hyperarousal subscale (of PTSD) which is characterized mainly by irritability, hypervigilance, difficulty concentrating, and heightened startle, had the highest correlation with Negative and Disorganized Schizotypy and the second-highest on Positive Schizotypy (slightly lower than the Intrusion subscale) (see Table 10). Hyperarousal is another sign of the mind's hyperactivity and being on alert, representing the relationship between attention deficit, hyperarousal, and Schizophrenic traits [e.g., see (121)].

The relationship between mental processes and personality traits was noted by Luria (122). Regarding how the imbalance in mental aspects may shape personality traits and behavior.

In addition to being dynamic, the system of meanings is also contradictory. One needs to grasp its internal contradictions to explain contrasting outcomes following the law of contradiction in psychomental development (78, 123–129)]. For instance, Positive Schizotypy exists alongside or is nourished by Negative Schizotypy. This becomes clear by noticing that Negative Schizotypy is higher, which may indicate that social withdrawal during the pandemic is associated with mental withdrawal (representing the internal dynamic of Negative Schizotypy), allowing the mind to trigger its defense mechanism to cope through the development of Positive Schizotypal traits [for the contradicted tendencies in schizophrenia one can see Vygotsky (120) as a classical reference]. In fact, by checking the answers of participants regarding how the pandemic affected their activities one can deduce that the majority reported that their activity was halted. Halting the activity means that the reported goals cannot be realized. Among participants who considered that the pandemic affected their activities, 45% reported that going out, shopping, and engaging in sports activities were affected, while another 46% considered that the activity, in general, is reduced or stopped (see Table 5). Also, by investigating the type of the goals, around 78% of participants reported that their goals had tangible aspects. This may be another factor that explains why when the activity was halted and disturbed, individuals tended to withdraw from social life, and hence tended to develop Negative Schizotypy. Indeed, there is a correlation between tangible goals and a higher level of Negative Schizotypy (see Table 12). Furthermore, results revealed a significant difference between participants who reported the existence of goals and those who did not in terms of the effect of the pandemic on activity. The existence of goals directing the activity revealed an association with the existence of the effect of the pandemic on the activity which means that individuals respond differently depending on how their activity system is structured (see Tables 8, 9). Going back to the weight of activity flow on Schizotypy, the effect of the pandemic contributed to the regression model regarding Positive Schizotypy but was not a predictor (see Table 11).

On other hand, different activities seem to have different associations with schizotypal traits, stress, and reflective function. For instance, engaging in sports and shopping is associated with lower Negative schizotypy. This may imply, in contrast to social withdrawal tendency, an association of socialization and attachment to the direct context with the low Negative Schizotypy. Also, both sport and shopping are associated with lower reflective function, which may imply that not reflecting on the context may decrease the ability to perceive the detachment from social context during the pandemic when the context is severely altered and being “vacuumed” from daily details. Indeed, both sports and shopping are negatively correlated with reflective function. The data revealed that high activity time is associated with lower reflective function. Also, it seems easier to compensate for shopping with online shopping and sports with the indoor sport as explained by participants. Our conclusion agrees with previous studies that more concrete goals are associated with less negative affect and distress, compared to abstract goals (109).

The above argument is supported by examining other sorts of activities. For instance, unlike tangible activities (e.g, sports and shopping), abstract activities are associated with a higher level of Positive and Disorganized schizotypy and high reflective function. This is the case of *doing research* (associated with Positive Schizotypy) and *volunteering* (associated with both Positive and Disorganized Schizotypy) since these two types of activities hold general socially-directed meanings and cannot be relatively compensated the same way as sports and shopping. Therefore, they will be mentally compensated through the inflation of certain schizotypal traits.

It seems that individuals who are not directed toward abstract meanings activities report low Reflective Function and low Negative Schizotypy (the case of *shopping* and *sports*), while individuals who are directed toward relatively abstract meanings tend to respond by developing Positive and Disorganized traits (the case of *doing research* and *volunteering*). Being engaged in *volunteering* activity may be associated with Disorganized Schizotypy by considering that *volunteering* structures the mind on a higher level than other sorts of tangible activity. Therefore, when volunteering is disturbed, the mind's structure will be disturbed as well by having higher Disorganized traits, and at the same time, it seems that the personality compensates for the loss of social engagement during volunteering events by the Positive Schizotypal traits. Also, the activity that transfers individuals into an *unreal* context is associated with Disorganized Schizotypy (in the case of *online games*). This is because the “narrative” that online games provide is not a part of the real-life narrative unlike other sorts of activities that have a connection with the ongoing life narrative.

These conclusions partially support the difference between genders by aiding to decrease the effect of gender imbalance in the sample size. For instance, males have significantly higher scores on online game activity, they belong to a one-child family more than females, and they are less engaged in shopping activities. Being a predictor of Positive Schizotypy, the one-child variable, in addition to that the males may have the potential feeling of being a *minority* among a majority of females in such Normal University types, supports the result that males scored higher on Positive Schizotypy. Being a minority (including sexual identity and gender minority) has a higher risk of developing Schizophrenic tendencies [e.g., see (130–133)]. Also, online games, in which males are engaged in higher than females are correlated positively with higher Disorganized Schizotypy (supporting the higher scores on disorganized schizotypy for male participants), and shopping is correlated negatively with Negative Shizotypy (males scored lower than females).

By reviewing CHAT's legacy including the functional method, compensation is a tendency of both the mind and brain, which is especially clear in the context of defectology [e.g., see (134)]. Also, compensation is mentioned in the writings of Oliver Sacks who is influenced by Luria [e.g., see (135–137)].

During lockdown and quarantine, the “life-term” goals became out of reach. So, due to the dynamic aspect of the meanings system, the pressure derived from the long period of halted activity may shift the goals implicitly and reconstruct the activity system. Therefore, to overcome the current unpleasant situation, the new goals related to pandemic-related lifestyle will gain more weight compared to the implicit reported goals that direct individuals' life. Therefore, even though 48% of participants reported having long-term goals, and 51% reported having medium-term aspects (2–3 years range), they are stained to more latent goals derived from social withdrawal. And one may argue that what is left is the mental compensation of the desired goals through schizotypal traits as discussed earlier.

In brief, due to the dynamic aspect of the meaning system forming the mind's structure, the organization of goals is not stable. Also, the personality and psychomental aspects will follow the continuous disruption in the activity flow, although individuals cannot explicitly grasp such re-organization of their system of meanings. This is the case when the reflective function is confronted with a disturbing context and faces the pressure of a current contextual impasse resulting from the disrupted life narrative. This may explain why high PTSD level was associated with all other variables regardless the difference in goals existence, goals type and term, motivation level, type of activity, and activity's time. The disturbance of life narrative was considered crucial in schizophrenia studies [e.g., see (138–141)].

Although it had a lower prediction value compared to other variables, PTSD was a predictor for three schizotypal traits' regression models. Also, the three subscales of PTSD (Intrusion, Avoidance, and Hyperarousal) were associated with the three schizotypy (see Tables 10–13) which goes with previous studies about the relationship between intrusion, avoidance, and hyperarousal on one hand, and schizophrenia on the other [e.g., see (121, 142–144)]. The association between high PTSD and high reflective function may reflect that people who consciously evaluate the disturbance of life's general narrative have more stress. Therefore, we may say that having a high level of reflective function is not always an element of lower psychopathological aspects. It depends on the meanings and the content being reflected. The linear regression model revealed that, in addition to the social relationship factor (the number of children in the family), the reflective function was found to be the strongest predictor of Positive and Disorganized Schizotypy.

**Figure 1:**
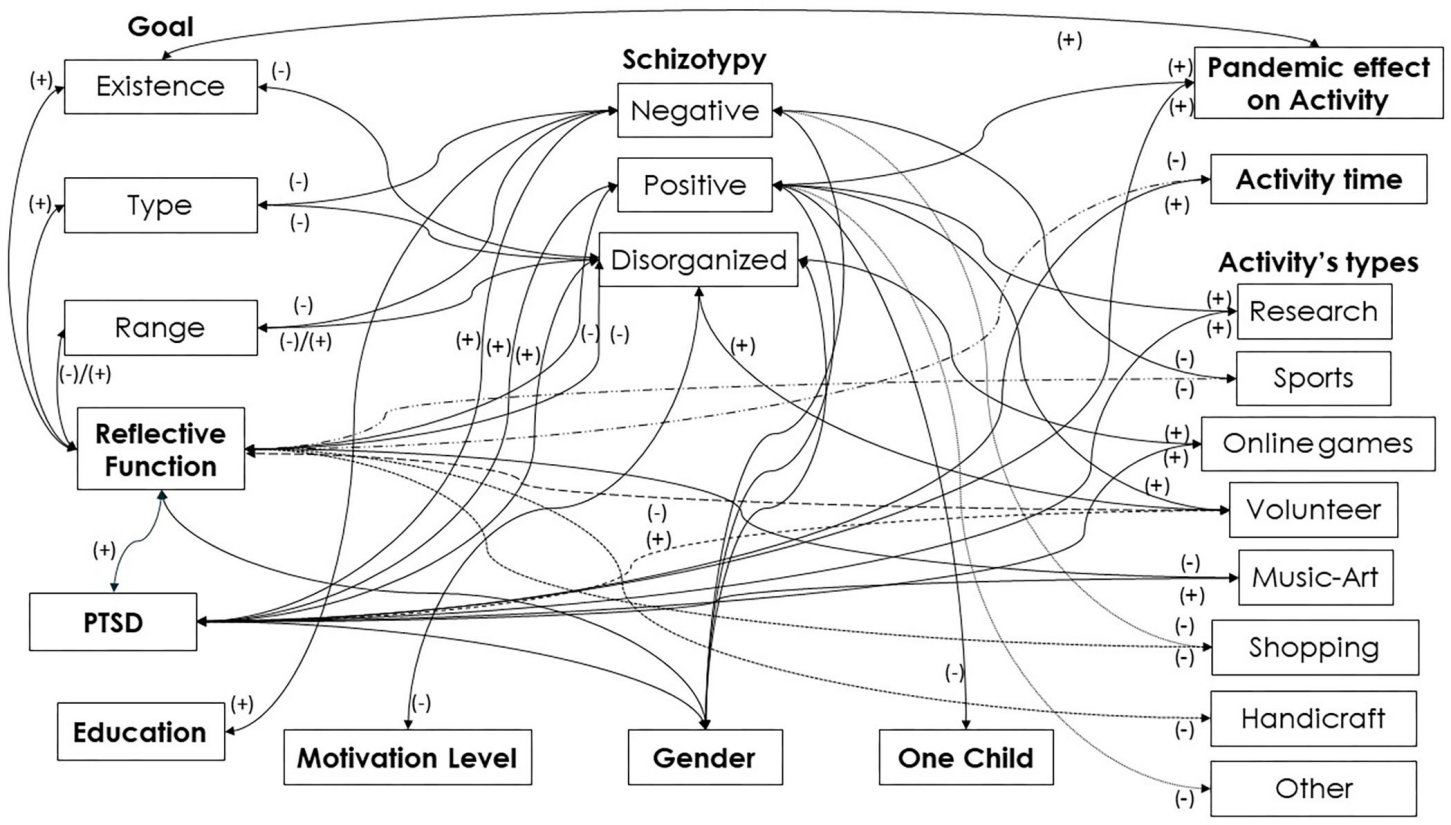
Variables' associations.

Overall, the same context may have numerous reflected meanings, which may be contrary for the same individual and may create contradictions in reading the outcomes. For a general representation of the associations among variables please see Figure 1.

## Conclusion

Overall, investigating the activity system and how it is changing (the internal conflicts according to the context) through time revealed a significant association with an extreme mind-state such as schizotypy (as a latent state of schizophrenia), especially in a social context where the life narrative is severely disrupted and is “on the edges.” So, the context of the study falls directly under the topic of thought disorder (TD) [e.g., see (145)]. To grasp how different individuals may experience the pandemic-related context (including restrictions and lockdown), some psychomental factors such as stress, anxiety, and depression, and other variables (such as media exposure, precautions, and knowledge about the pandemic) are required to be investigated. It is crucial to analyze the disturbance and the changes in the dynamic system of meanings and the flow of thought both in space and time. Being able to draw a dynamic view about how the system of meanings and thoughts may change in the pandemic context (and any other similar situation when the reality is being sharply disrupted) according to the general change of activity system, one can increase individuals' ability to cope and to gain the required psychomental tools in facing critical psychomental health problems. Building on Vygotsky's guidelines, some studies noted that cognitive flexibility is negatively correlated with psychopathology which we think is required in a critical fast-changing context like the one during a pandemic. Cognitive flexibility is required, especially regarding goal formulations and planning under the label of executive function [e.g., see, (119)].

Furthermore, according to the study results, future work needs to be performed regarding the elaboration of an intervention protocol not only for pandemic-related lifestyles but also for other “living on the edges” contexts. The protocol's role is to provide a *space-time* structure, such as the introduction of dynamic planning of activities according to individuals' interests and goals along with the continuous changes in the context. In addition, the protocol should include the development of mental skills such as mentalization and reflection in line with the content of the conceptualization and themes investigated in this study. In other words, in addition to the design and proposal of activity structure in general, the protocol should provide the participants the ability to develop self-reflective skills using the *narrative, goals description, activity changes and context, motivation sources, etc*. as tools to control and plan their own behavior in line with the dynamic and continuous changes in the situation.

Future studies need to be conducted for in-depth investigations about how the life narrative is disrupted. Additionally, they need to investigate how different individuals reflect on the disruption. This is necessary when the current disturbance in the world view, especially during the last decade, goes beyond the context of the pandemic, and when the “modern” lifestyle and life narrative are being altered around the world due to the socio-economic and cultural crisis.

## Data availability statement

The original contributions presented in the study are included in the article/supplementary material, further inquiries can be directed to the corresponding author.

## Ethics statement

The studies involving human participants were reviewed and approved by Committee of Academic Ethics of Zhengzhou Normal University. The patients/participants provided their written informed consent to participate in this study.

## Author contributions

ME: conceptualization, analysis, and writing. ME and YW: data interpretation. YW: revising intellectual content. ME and ZJ: methodology. KZ and TT: data collection. KZ: translation. ME and TT: encoding. YL: editing. YW: providing financial support that are necessary for this study. All authors contributed to the manuscript final form. All authors contributed to the article and approved the submitted version.

## Funding

This study was partially supported by Program for Science and Technology Development of Henan Province (222102310686) and Talents Program of the Ministry of Science and Technology of the PRC. And partially supported by YW, University of Sheffield.

## Conflict of interest

The authors declare that the research was conducted in the absence of any commercial or financial relationships that could be construed as a potential conflict of interest.

## Publisher's note

All claims expressed in this article are solely those of the authors and do not necessarily represent those of their affiliated organizations, or those of the publisher, the editors and the reviewers. Any product that may be evaluated in this article, or claim that may be made by its manufacturer, is not guaranteed or endorsed by the publisher.
